# Molecular and Serological Evaluation of Toxoplasma gondii Infection in Reared Turkeys in Fars Province, Iran

**DOI:** 10.5812/jjm.11598

**Published:** 2014-07-01

**Authors:** Bahador Sarkari, Qasem Asgari, Neda Bagherian, Soheil Ashkani Esfahani, Mohsen Kalantari, Iraj Mohammadpour, Majid Ashrafmansori, Maryam Amerinia, Fatemeh Sabet Sarvestani

**Affiliations:** 1 Basic Sciences in Infectious Diseases Research Center, Shiraz University of Medical Sciences, Shiraz, IR Iran; 2 Department of Parasitology and Mycology, School of Medicine, Shiraz University of Medical Sciences, Shiraz, IR Iran; 3 Student Research Committee, Shiraz University of Medical Sciences, Shiraz, IR Iran

**Keywords:** Toxoplasma, Polymerase Chain Reaction, Biological Assay, Turkeys

## Abstract

**Background:**

Toxoplasmosis is a parasitic disease caused by the protozoan *Toxoplasma gondii*. This parasite infects most of warm-blooded animals, including birds. Turkeys are one of these animals which might be infected by this parasite. Little is known about the prevalence of *T. gondii* in turkeys in Iran.

**Objectives:**

The current study aimed to evaluate the rate of *Toxoplasma* infection in turkeys in Fars Province, Southern Iran.

**Materials and Methods:**

Sera and tissues (brain, neck and tongue) of 54 turkeys were collected from Shiraz slaughterhouse in Fars province. Anti-*Toxoplasma* antibodies were assessed in the collected sera using modified agglutination test (MAT), while tissues were evaluated by polymerase chain reaction (PCR) and bioassay methods.

**Results:**

*T. gondii* antibodies (MAT titer: ≥ 1:40) were found in 89.8% of turkeys. *T. gondii* DNA was detected in 61.6% of turkey tissues and brain had the highest rate of infection. Brain tissues from each animal were bioassayed and *Toxoplasma* tissue cysts were found in 11.5% and *Toxoplasma* DNA in 62% of inoculated mice.

**Conclusions:**

Results of this study validated a relatively high level of *Toxoplasma* infection in reared turkeys and turkey meat might be considered as an infection sources for human.

## 1. Background

*Toxoplasma gondii* is a protozoan which can infect humans and warm-blooded domestic and wild animals including birds. Humans are commonly infected with *T. gondii* by consumption of oocysts present in soil or water, polluted with cat feces, or ingestion of tissue cysts in underdone meat (1-3). Nevertheless, transmission of *T. gondii* is more likely through ingestion of infected tissues than ingestion of oocysts (1).

The infection is particularly important in women when they acquire the infection for the first time during their pregnancy, where an intrauterine transmission of the parasite may occur. Congenital toxoplasmosis affects 1-10 per 10000 infants in Europe. Its incidence and severity vary depending on the time of infection in mothers. Transmission rate increases with gestational age, whereas the severity of infection reduces by the time of infection. Food animals including pigs, sheep, goats, and reared birds, such as chickens and turkeys, can be infected by *Toxoplasma* and these animals can transmit the infection to humans through their meats. Birds are important intermediate hosts of *Toxoplasma,* since they serve as a source of infection for humans as well as an important reservoir for cats (4). Human toxoplasmosis is a common protozoan infection in Iran (5).

Seroprevalence rate of toxoplasmosis in general population of Iran has been reported 51.8% (5). This rate varies in different parts of the country. Seroprevalence rates of 55% and 29% have been reported for toxoplasmosis in northern and southern parts of Iran, respectively. In Fars province, southern Iran, seroprevalence rate of toxoplasmosis in sheep and goats have been reported 26.5% and 14.02%, respectively (6). *Toxoplasma* infection in human occurs commonly through consumption of undercooked or raw meat. Infected lamb meat is considered as one of the main sources of *Toxoplasma* infection in human (7). In a recent study, *Toxoplasma* infection in edible tissues of sheep and goats in Fars province was evaluated by molecular methods. The study revealed that five of 22 goats (22.7%) and 21 of 56 sheep (37.5%) were infected by *Toxoplasma* (8). Cats are the final hosts for *Toxoplasma* and rate of infection in these reservoir hosts has been relatively high in Iran (9). In the recent years, breeding of turkeys has been quite common in Iran, including Fars province. Meat of these birds might be infected by *Toxoplasma* and transfer the infection to humans. Prevalence of *Toxoplasma* infection in turkeys varies in different parts of the world, ranging from ≤ 1% to 80% (10-12).

## 2. Objectives

The current study evaluated seroprevalence of *T. gondii* in reared turkeys in Fars province, southern Iran. Moreover, the study aimed to assess the rate of *Toxoplasma* infection using molecular methods in animal tissues. The study was justified by the lack of information about prevalence of *T. gondii* in turkeys in Iran.

## 3. Materials and Methods

### 3.1. Study Area and Sample Collection

The current study was conducted in 2012 in Fars province, southwest of Iran Farms for rearing free-range turkeys have been growing during the last decades in this region. Sera and tissue samples (brain, neck muscle and tongue) were collected from 54 turkeys from slaughterhouse in Shiraz from April to September 2012. Collected sera were evaluated for anti-*Toxoplasma* antibodies by modified agglutination test (MAT). Tissue samples were assessed for *Toxoplasma* infection by PCR and bioassay methods.

### 3.2. Modified Agglutination Test

MAT was performed using formalin-fixed whole RH tachyzoites, as originally explained by Desmonts and Remington (13). Briefly, the test was performed, using formalin-fixed *Toxoplasma* tachyzoites. Turkeys and mice serum samples were diluted with phosphate buffered saline (PBS, pH = 7.2), and evaluated for anti-*Toxoplasma* IgG antibodies. The antigens were diluted with antigen diluting buffer, containing bovine serum albumin (BSA), 2-mercaptoethanol and Evans blue dye solution. Positive and negative sera, as controls, were incorporated in each plate with same dilutions as the test sera. Sera with MAT titers of 1:40 or higher were considered as positive. A blue pellet at the base of the U-shape-well microtiter plates (Nunc) were considered as negative, while agglutination of the formalin-fixed parasite in a mat, covering about half of the well base, was recorded as positive.

### 3.3. Detection of T. gondii in Turkey Tissues by PCR

DNA was extracted from 1 g of the homogenized tissues. Proteinase K and lysis buffer (1 mM of EDTA, pH = 8.0; 50 mL of Tris–HCl, pH = 7.6; 1% of Tween 20) were added to the samples and incubated at 37°C for 24 hours. Phenol/chloroform/isoamyl alcohol was used to extract the lysate, and the DNA was precipitated using absolute ethanol (8). Nested primer sets (Bioneer, Korea) were used to amplify the fragments of the B1 gene, as previously reported (8). Briefly, outer primers (5´-CCG TTGGTT CCG CCT CCT TC-3´ and 5´-GCA AAA CAG CGG CAGCGT CT-3´), producing an amplified product of 432 bp and inner primers, (5´-CCG CCT CCT TCG TCCGTC GT-3´ and 5´-GTG GGG GCG GAC CTC TCT TG-3´), producing an amplified product of 213 bp were used. Details of the PCR program are given in Tables 1 and 2.

**Table 1.** Details of Nested-PCR Program (first step) for Amplification of *Toxoplasma* B1 Gene

Program Toxo 1	Segment	Definition	Temperature, °C	Time, min	No. of Cycles
**Program 1**	1	Heat start (amplification)	94	2	1
**Program 2**					
	1	Denaturation	94	1	30
	2	Annealing	57	2	30
	3	Extension	72	3	30
**Program 3**	1	Final	72	10	1
**Program 4**	1	-	4	99/99	-

**Table 2.** Details of Nested-PCR Program (Second Step) for Amplification of *Toxoplasma* B1 Gene

Program Toxo 1	Segment	Definition	Temperature, °C	Time, min	No. of Cycles
**Program 1**	1	Heat start (amplification)	94	2	1
**Program 2**					
	1	Denaturation	94	1	35
	2	Annealing	58	1	35
	3	Extension	72	1	35
**Program 3**	1	Final	72	10	1
**Program 4**	1	-	4	99/99	-

### 3.4. Bioassay in Mice

Brain tissues of turkeys were inoculated intraperitoneally into mice. All the mice were monitored daily over two months. After that, blood samples were taken from the mice for serological assays and their brains were removed for cyst detection and DNA extraction. Impression smears were prepared from livers, spleens and brains of mice for detection of tachyzoites or tissue cysts. Tissues of inoculated mice were examined for *Toxoplasma* DNA by PCR.

### 3.5. Statistical Analysis

All statistics were performed using SPSS version 16 statistical package. Chi-square test was used to determine the influence of examined factors on seropositivity to *Toxoplasma*. P value less than 0.05 was considered statically significant. Degree of agreement was quantified by kappa statistic.

## 4. Results

*T. gondii* antibodies (MAT titer: ≥ 1:40) were found in 89.8% of turkeys. PCR detected 351- and 546-bp bands, corresponding to *Toxoplasma* infection, in tissues of 33 of 54 (61.6%) turkeys. Brain was the most infected tissue (33.3%), followed by tongue (31.5%) and neck (29.6%) tissues (Table 3). Brain tissues from each animal were bioassayed and *Toxoplasma* tissue cysts were found in 11.5% and *Toxoplasma* DNA in 62% of inoculated mice. Coinfection of brain and neck was noticed in eight, brain and tongue in 12, and neck and tongue in eight turkeys. MAT was positive in 16 PCR-negative animals and was negative in three PCR-positive cases. The agreement between PCR and MAT was 62%. Statistical analysis of MAT and PCR findings showed that there were no significant correlation between seropositivity and PCR positivity of brain, neck and tongue tissues. Results of bioassay in mice revealed tissue cysts in brain of 11.5% of the inoculated mice, while PCR detected *Toxoplasma* infection in brain of 31 of 50 (62%) inoculated mice.

**Table 3.** Molecular and Serological Rate of *Toxoplasma* Infection in Reared Turkeys in Southern Iran ^a^

PCR	Neck		Brain		Tongue		Total	
MAT	Positive	Negative	Positive	Negative	Positive	Negative	Positive	Negative
**Positive**	9	24	11	22	8	25	27	16
**Negative**	1	6	2	5	2	5	3	4

^a^ Abbreviations: MAT, modified agglutination test; PCR, polymerase chain reaction.

## 5. Discussion

Nearly one third of the world population is infected by *Toxoplasma* and consumption of underdone meat is considered as the main causes of infection (2). The disease is mainly asymptomatic in immune-competent individuals and clinical signs may be present in few of the infected people (1). *Toxoplasma* infection is common in various animals including sheep, pigs, goat, and poultry, which are used for food. Infection in other animals such as cattle, horses, and buffaloes is less prevalent. Domestic turkeys are usually resistant to clinical toxoplasmosis (4). Results of experimental infections demonstrated that even one or two-week-old turkeys inoculated with millions of tachyzoites of RH strains became infected, but did not develop clinical signs, while all of experimentally infected turkeys harbored *T. gondii* in their tissues (14). Similar results were obtained by feeding *T. gondii* oocysts to turkeys, where turkeys were fed with oocysts and viable *T. gondii* were recovered from tissues of all turkeys by bioassays in mice and all the turkeys developed antibodies to *T. gondii*, detectable by MAT (15).

In this study, we used MAT for detection of anti-*T. gondii* antibodies in the reared turkeys or mice. MAT is based on detection of IgG antibodies, produced by the host against *Toxoplasma,* and uses formalin-fixed *Toxoplasma* tachyzoites. MAT is an appropriate test for seroprevalence studies of toxoplasmosis in animals since it does not need species-specific conjugates usually used in IFA or ELISA methods. Sensitivity and specificity of MAT has been reported 82.9% and 90.29%, respectively, in comparison with other commercially available serological assays for detection of *Toxoplasma* IgG antibodies in animals. MAT is a method extensively used for seroprevalence studies in the recent years (16-21).

Findings of this study revealed that more than 80% of reared turkeys in southern Iran were infected with *Toxoplasma*. Comparing the rate of *Toxoplasma* infection in this study with currently available reports from different geographical areas, higher prevalence of *Toxoplasma* in turkeys can be seen in this area (10, 11, 22, 23). In Germany, seroprevalence of *T. gondii* in 1913 turkeys from 14 turkey farms from different areas of the country was 20.2% (ranging from zero to 77.1%) (10). An antibody prevalence of 10% has been reported in turkeys of the southeastern United States (22). In another study, anti-*Toxoplasma* antibodies were detected in 12 of 17 sera from turkeys of Alabama (12). In Egypt, *T. gondii* seroprevalence was examined in turkeys, showing prevalence rate of 29.4% (11).

High level of seropositivity in our study, compared with other studies, might be linked to high level of *T. gondii* infection in animal and also human in our region. In a recent study, conducted by the author of the current study, seroprevalence of toxoplasmosis in farm animals was evaluated. Sera from farm animals including cows, dogs, horses, sheep and goats, were serologically assessed for anti-*Toxoplasma* antibodies. Result showed that 121 of 346 (34.9%) animals were infected by *Toxoplasma*. Cattle had the highest rate of infection, followed by dogs, horses, sheep and goat (24).

Raeghi et al. evaluated seroprevalence of toxoplasmosis in Urmia, capital of West Azerbaijan province, Iran, and found that 21.1% of sheep, 1.6% of cattle, and 11.5% of horses were seropositive for *T. gondii*. They also reported that sheep were 15 times more likely to be seropositive for *Toxoplasma,* compared with cattle (25). Tissue cysts of *T. gondii* may last in food animals for years. Almost all edible portions of an animal can comprise viable *T. gondii*. Rate of *T. gondii* tissue cysts in meat of food animals is usually low. In 100 grams of meat, as few as one tissue cyst may be present; thus, it is difficult to detect this low level of *T. gondii* infection in tissues of animals, unless a reliable molecular approach is used.

*T. gondii* can be isolated from animals by inoculation of laboratory animals with animal tissues. Microscopical detection of *Toxoplasma* tissue cyst in tissues of animals is not a reliable method. Moreover, histochemical or immunohistochemical staining of parasites are not sensitive methods for detection of *T. gondii* in animal tissues (26). In several studies, detection of *T. gondii* DNA in meat of animals or human tissues by molecular approaches has been described (8, 24, 27, 28). However, efficacy of these methods has not appropriately been defined. Improved methods such as real-time PCR and fluorogenic probe can detect DNA of as few as four bradyzoites of *T. gondii* in tissues or other samples of humans or animals. *T. gondii* isolates of human and animal sources are clustered into three clonal types, I, II and III (29). Features of these lineages are different in a variety of aspects including their virulence in laboratory animals. While infection with type I is lethal for outbred mice, infections with types II and III are usually less virulent in these hosts. Type II strains are the main common types of *Toxoplasma* in North America and Europe.

Prevalence and genotypes of *Toxoplasma* in avian hosts (free-range chickens, sparrows, pigeons and starlings) in the southwest of Iran (Khuzestan province) has been evaluated by Khademvatan et al. (19). The results demonstrated that 16.5% of samples of avian tissues were infected with *T. gondii*. Molecular typing and DNA sequencing found types II and III *T. gondii* as the predominant lineages of the parasites. Moreover, a higher prevalence rate of type III, compared with type II, was reported in their study (19). Results of the current study were in accordance with those showing a high prevalence of *Toxoplasma* in this region in animals and humans.

Brain was the most infected tissue in our study, followed by neck muscle and tongue. Although seropositivity of turkeys is not directly linked to infection of tissues, however, results of bioassays in mice demonstrated that tissues of seropositive turkeys can induce infection in mice. It should be highlighted that seropositivity of turkeys to *Toxoplasma* does not necessarily mean the infectivity for consumers, except for birds with positive bioassay results. Supporting this point, some seropositive turkeys were PCR-negative. Considering the high rate of *T. gondii* infection in turkeys in the region, undercooked eatable tissues of these birds must be considered as sources of *Toxoplasma* infection for humans in the region. Proper cooking of turkey meat can be recommended for preventing the transmission of *Toxoplasma* to human. Moreover, not feeding raw or undercooked meat to cats and keeping cats outside the turkey breeding farms will reduce the risks of infection from cats to turkeys.
